# Modeling and forecasting of at home activity in older adults using passive sensor technology

**DOI:** 10.1002/sim.9529

**Published:** 2022-07-20

**Authors:** Jess Gillam, Rebecca Killick, Jack Heal, Ben Norwood

**Affiliations:** ^1^ STOR‐i Doctoral Training Centre Lancaster University Lancaster UK; ^2^ Department of Mathematics and Statistics Lancaster University Lancaster UK; ^3^ Howz Greenheys Business Centre Manchester UK

**Keywords:** autoregressive, binary series, home sensing

## Abstract

Life expectancy in the UK has increased since the 19th century. As of 2019, there are just under 12 million people in the UK aged 65 or over, with close to a quarter living by themselves. Thus, many families and carers are looking for new ways to improve the health and care of older people. Passive sensors such as infra‐red motion and plug sensors have had success as a noninvasive way to help the older people. These provide a series of categorical sensor events throughout the day. Modeling this categorical dataset can help to understand and predict behavior. This article proposes a method to model the probability a sensor will trigger throughout the day for a household whilst accounting for the prior data and other sensors within the home. We present our results on a dataset from Howz, a company helping people to passively identify changes in their behavior over time.

## 1 INTRODUCTION

Life expectancy in the UK has increased since the 19th century.^1^ As of 2019, there are just under 12 million people in the UK aged 65 or over,^2^ with close to a quarter living by themselves. Those living on their own are more prone to trips to the emergency room and are more likely to suffer from mental illnesses.^3^ An ageing population such as this results in extra pressures for the NHS and health care services. By 2030, it is likely that one in five people will be aged 65 or over.^4^ It is clear there is a growing need for new ways to help the ageing population and reduce strain on the NHS and health care services.

### 1.1 Motivation

Howz is a product which introduces a number of nonintrusive sensors within the homes of older people and care facilities. The data received from these sensors are used to understand a household's behavior, which can be used to improve the health and well‐being of the household. For example, if a customer starts getting up during the night more frequently it could indicate a water infection. Once Howz has detected this, they make the user, family member or carer aware, so that action can be taken, reducing severity and preventing hospitalization.

In this article, we focus on estimating the probability a sensor will trigger in a 15‐minute interval given the other household sensors. We look at this information on a 24‐hour scale, as previous analysis^5^ indicates a household is likely to follow similar routines throughout the day. For example, in the process of getting up we could expect the bedroom door, bathroom door and kettle sensor. Therefore, if the bedroom and bathroom sensors are triggered, we would expect an increase in the estimated probability of the kettle sensor.

#### 1.1.1 Howz anonymized dataset

**FIGURE 1:**
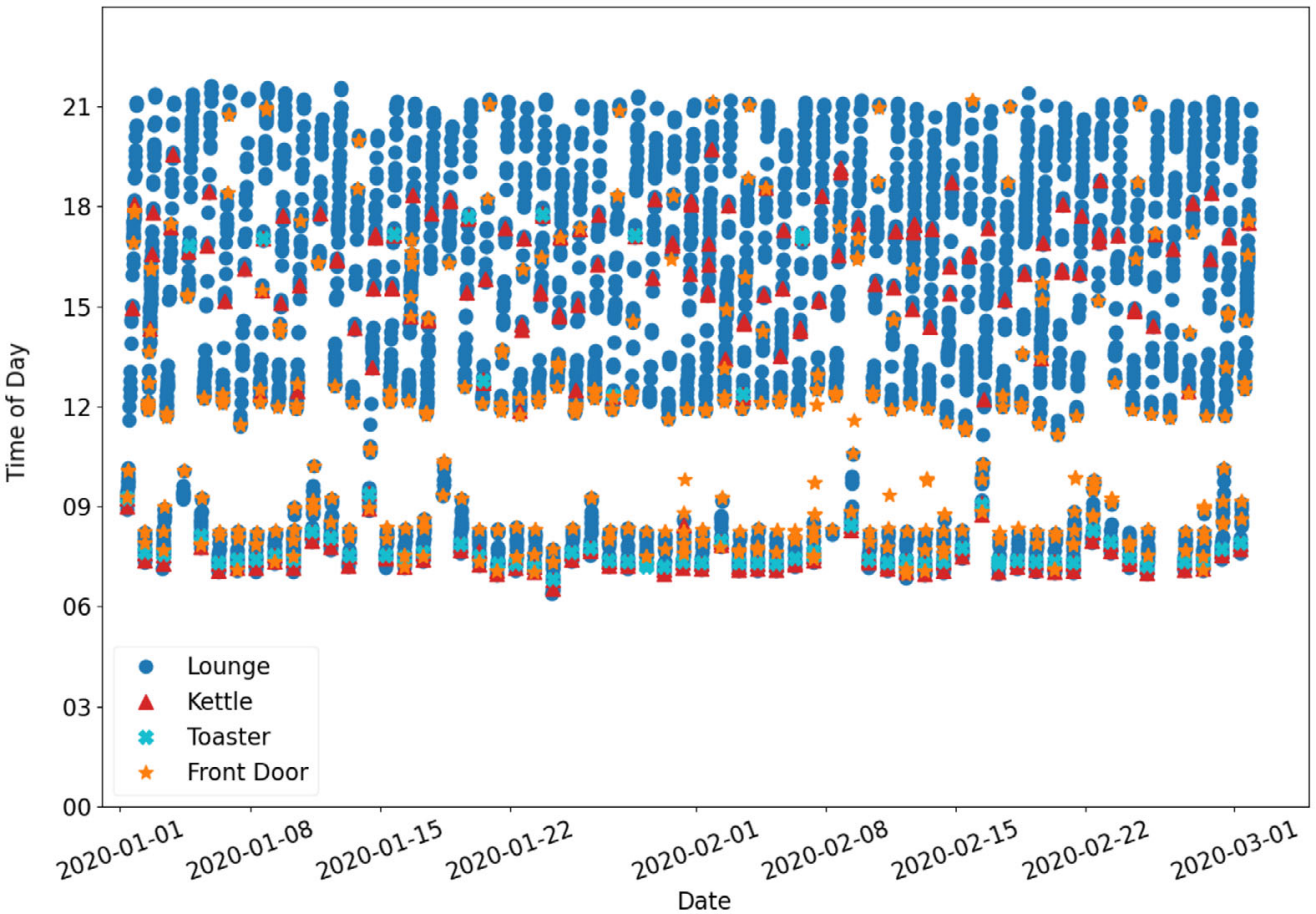
Color‐coded sensor events for a household: Lounge, toaster, kettle, and front door

Figure 1 is an example of data for a household with four sensors: lounge, toaster, kettle, and front door. Howz typically provide three sensors as a starter package for a new customer, with the option to install additional sensors. It is the customer who chooses how many sensors are used with some households having up to 10 sensors. Each data point has a sensor type and whether the sensor was triggered within the 15‐minute interval.

**FIGURE 2:**
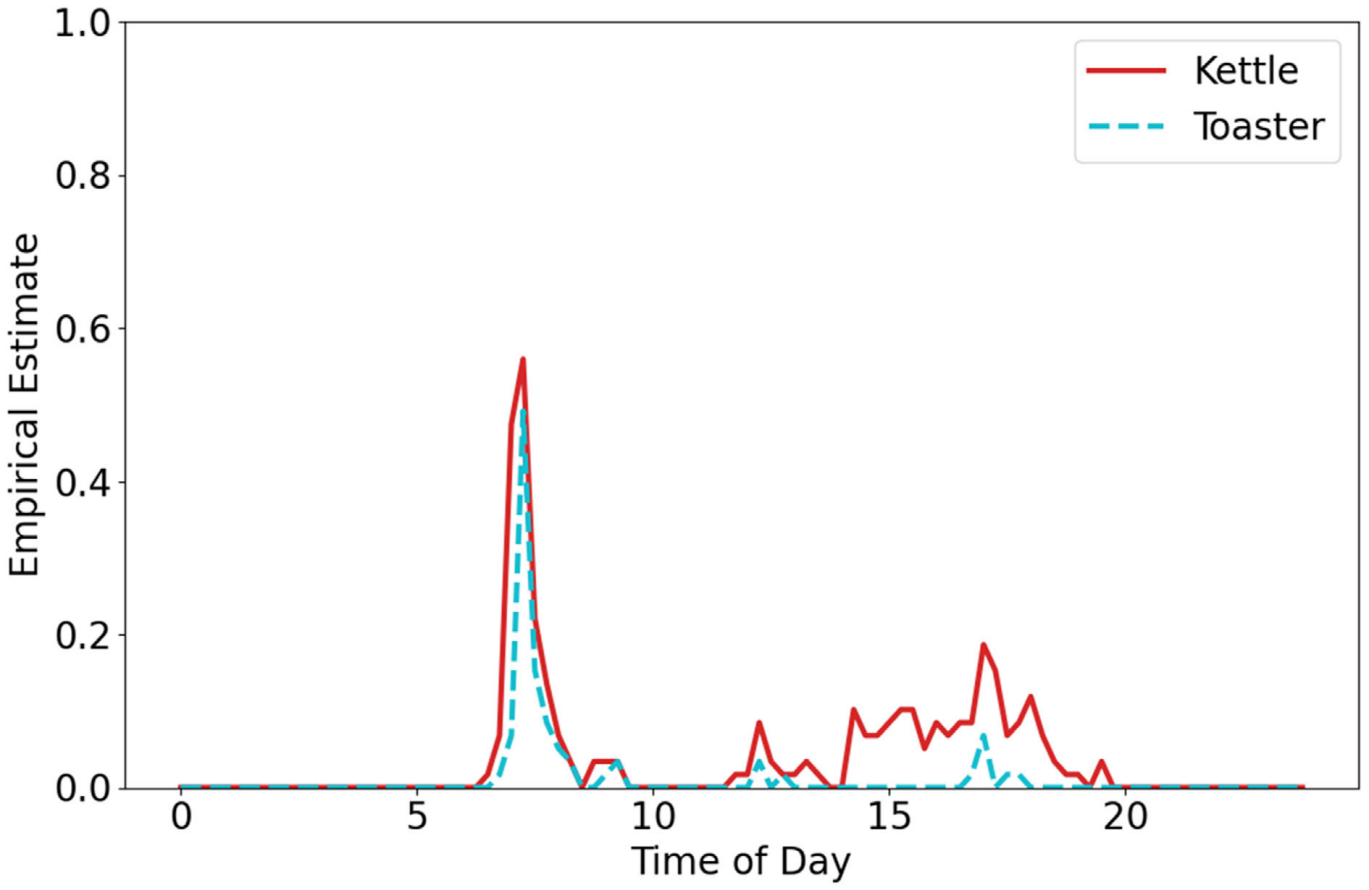
Empirical probability of the kettle sensor triggering at each time interval (red line) vs toaster sensor (blue)

We are interested in presenting the data on a 24‐hour scale to explore how the household changes over the day. Figure 2 shows an example of this empirical probability in February for the kettle and toaster sensors. From the empirical estimate, we can see that the probability varies throughout the day with the highest between 6 and 9 am and the kettle having low level activity in the afternoon until 8 pm with very little activity for the toaster after 9 am.

### 1.2 Statistical motivation and structure

For discrete time series data, the main body of research concerns time series analysis of count data.^6^ See *Handbook of discrete‐valued time series* ^7^ for a general book on methods for discretized data. However, for our application it is necessary to look at the data within the day, rather than the daily count, and how that changes day to day. For example, using kitchen sensors at different times within the day could be a sign of forgetting to eat until the user becomes very hungry. Households tend to follow small routines throughout the day, that is, getting up and getting ready for the day and getting ready for bed. Hence, particular sensors are likely to be seen in sequence with each other. There has been recent research into classifying such routines with the use of wearable technology and other sensors with notable research papers gathered in Lotfi et al.^8^ In contrast, our focus is on incorporating potential routines into the model, not identifying them, so we look to alternative methods. Giving device specific feedback to the user, especially if the family or carers have specific concerns is very important. Therefore, we view the data as multiple binary time series. We acknowledge there are many ways of viewing the data, however, this best fits Howz's goal and provides more specific information to relay to customers. Typical generalized linear models for binary data often use the Bernoulli distribution with the logit link function. Examples in the literature include; a Bernoulli autoregressive moving average model^9^ and analyzing eye tracking data with multilevel logistic regression.^10^ However, to accurately estimate the probability, the method must be able to follow the steep spikes and dips seen in the data (Figure 2), which suggests classic linear modeling will not suffice. As indicated above, the current literature, contains time series models with autoregressive components. An alternative approach is to use Hawkes Processes. Hawkes processes place emphasis on using the recent data to inform the current probability on data that is more prone to steep spikes. Engelhard et al^11^ use Hawkes processes for predicting smoking events and Jewell et al^12^ for identifying spikes in calcium imaging data.

We propose a new model specification for modeling a sensor that takes inspiration from Hawkes processes within a generalized linear framework. It will dynamically vary the probabilities that a sensor will be triggered throughout the day, based on the prior data from the sensor itself and the sensors around it to incorporate the effect of routines in the data. In Section 2, we present the method, and in Section 3, we apply it to Howz data. In Section 4, we compare this method to a logistic regression model, and in Section 5, we summarize the results and discuss potential avenues for further research.

## 2 MODEL

The following method considers each sensor separately and models the behavior as a binary time series conditional on the data up until the previous time point. Recall the data is sampled every 15 minutes, where T is number of data samples we have. Similar to the set up in Fokianos and Moysiadis,^13^ and Milani et al,^14^ let $\left\{Y_{t}:t=1,\ldots ,T\right\}$ be a binary time series, the population random variable we are seeking inference upon. Then let $y_{t}$ be the observed binary outcome for the sensor of interest at time t:

$$y_{t}=\left\{\begin{array}{ll} 1,\; & \text{if the sensor of interest is triggered within the 15 minutes immediately prior to time}\;t,\\ 0,\; & \text{otherwise.} \end{array}\right.$$

Note that, if the sensor is triggered multiple times in the 15 minutes this information is not included.

Let $F_{t-1}$ be the information we have until time t,

$$F_{t-1}=\{y_{t-1},\ldots ,y_{1},z_{j,t-1},\ldots ,z_{j,1},p_{t-1},\ldots ,p_{1}\},$$

where $y_{i}$ for i<t from the modeled sensor. We let J denote the number of other sensors and $z_{j,i}$ be the other sensors j∈J at time i<t and prior expectations $p_{t}=E(Y_{t}|F_{t-1})$. The conditional distribution for each $Y_{t}$ is modeled as,

$$Y_{t}|F_{t-1}∼\text{Bernoulli}(p_{t}).$$

Using a generalized linear model framework, we estimate the conditional mean, $p_{t}$, which is the probability that $Y_{t}=1$ at time t given the information up until time t. In order to assess whether there has been a change in behavior, the previous information for a households routine is very important.

Motivated by our application, we now consider the form of the linear predictor, $\Delta_{t}$. For the Bernoulli distribution, a natural choice for a canonical link function is the logit function.

$$\Delta_{t}=\text{logit}(p_{t})=\log\left(\frac{p_{t}}{1-p_{t}}\right).$$

This maps the linear predictor using the link function from (−∞,∞) to (0,1) for the estimated probability $p_{t}$. Due to the placement of sensors throughout the house, they will often be triggered in a certain order, that is, to get from the bedroom to the bathroom, a member of the household must go through the hallway. We would also expect certain sensors to have some dependence on when it was last seen. For example, the hallway could be triggered before entering the bathroom and exiting, or if the kettle is used we may not expect to see the kettle again for a few hours. Therefore, $\Delta_{t}$ should contain an autoregressive term $b_{t}$, a dependence on other sensors $c_{j,t}$ and a seasonal factor $d_{t}$. We include a seasonal factor due to sensors often being seen at similar times days previously. For example; putting on the kettle and having a morning coffee before leaving for work.

$$\Delta_{t}=a+b_{t}+\overset{J}{\underset{j=1}{\sum }}c_{j,t}+d_{t}.$$

Moysiadis and Fokianos^15^ let $b_{t}=\eta b_{t-1}+\nu y_{t-1}$ where η,ν∈ℛ. However, due to the steep changes in probability, see Figure 2, we need to place more restrictions on η. Instead we take inspiration from Jewell et al^12^ who include a rate parameter, η, that decays exponentially. In the context of the sensor dataset, we would want a spike to occur for $b_{t},c_{j,t}$, and $d_{t}$ based on the previous time point. For this application, this could be in a negative or positive direction depending on the sensor, that is, if a kettle sensor is triggered we may not expect the next one for a few hours, however a lounge sensor might be triggered a lot if the household spends most of the day there. Take $b_{t}$ such that

$$b_{t}=\phi_{b}b_{t-1}+\pi_{b}y_{t-1},$$

where $\pi_{b}\in ℛ$ is the spike in the estimate. The equation will decay exponentially at a rate governed by $\phi_{b}$ where $\left|\phi_{b}\right|<1$ to incorporate positive and negative rates of decay. We express the other sensors similarly,

$$c_{j,t}=\psi_{j}c_{j,t-1}+\tau_{j}z_{j,t-1},\;\text{for sensors}\;j=1,\ldots ,J.$$

For the seasonal term, $d_{t}$, we would like to acknowledge that routines can vary in their timing. Thus we have allowed 15 minutes either side of the time the previous day (45‐minute window),

$$d_{t}=\phi_{d}d_{t-96}+\pi_{d}\max(y_{t-96-1},y_{t-96},y_{t-96+1}).$$

The term $y_{t-96}$ signifies the same time the day previously, due to having 96 points a day as the data are sampled every 15 minutes. With this formulation of $\Delta_{t}$ we have,

$$p_{t}=\frac{\exp(a+b_{t}+\overset{J}{\underset{j=1}{\sum }}c_{j,t}+d_{t})}{1+\exp(a+b_{t}+\overset{J}{\underset{j=1}{\sum }}c_{j,t}+d_{t})}.$$

Now we have defined $p_{t}$, we can simplify for ease of parameter estimation. We are able to simplify the link function in the following proposition.

Proposition 1 *We can simplify* $b_{t}$ *to*

$$b_{t}=\pi_{b}\overset{t-1}{\underset{i=1}{\sum }}\phi_{b}^{i-1}y_{t-i}.$$

*Similarly,*

$$c_{j,t}=\tau_{j}\overset{t-1}{\underset{i=1}{\sum }}\psi_{j}^{i-1}z_{j,t-i}.$$

*For the seasonal term, considering* t≥96 *,*

$$d_{t}=\pi_{d}\overset{\left\lfloor \frac{t}{96}\right\rfloor }{\underset{i=1}{\sum }}\phi_{d}^{i-1}\max(y_{t-96i-1:t-96i+1}),\;\text{for}\;t\geq 96.$$

*Therefore,* $\Delta_{t}$ *and* $p_{t}$ *can be expressed as,*

$$\begin{array}{llll} \Delta_{t} & =a+\pi_{b}\overset{t-1}{\underset{i=1}{\sum }}\phi_{b}^{i-1}y_{t-i}+\overset{J}{\underset{j=1}{\sum }}\tau_{j}\overset{t-1}{\underset{i=1}{\sum }}\psi_{j}^{i-1}z_{j,t-i}\; & & \\ & \;+\left\{\begin{array}{cc} \pi_{d}\overset{\left\lfloor \frac{t}{96}\right\rfloor }{\underset{i=1}{\sum }}\phi_{d}^{i-1}\max(y_{t-96i-1:t-96i+1}),\; & \text{if}\;t\geq 96,\\ 0,\; & \text{otherwise,} \end{array}\right.\; & & \\ p_{t} & =\frac{\exp(\Delta_{t})}{1+\exp(\Delta_{t})}.\; & & \end{array}$$

*For a detailed proof, see Appendix* *A*.

We estimate the parameters using the maximum likelihood.

Proposition 2 *Let*

$$\alpha =\{a,\pi_{b},\phi_{b},\tau_{1},\psi_{1},\ldots ,\tau_{j},\psi_{j},\pi_{d},\phi_{d}\},$$

*be the parameters we wish to estimate. Then we can formulate the log‐likelihood as,*

$$l(\alpha |{\left\{y_{t}\right\}}_{t=1}^{T})=\overset{T}{\underset{t=1}{\sum }}y_{t}\Delta_{t}\;-\;\overset{T}{\underset{t=1}{\sum }}\log\left(1+\exp(\Delta_{t})\right).$$

*For the full derivation, see Appendix* *B*.

Due to the form of $\Delta_{t}$, analytical expressions for $\hat{\alpha }$ are not attainable. Thus to estimate $\hat{\alpha }$, we use a common numerical optimization method called Broyden‐Fletcher‐Goldfarb‐Shanno (BFGS), see Fletcher.^16^ This optimization method and versions of it are widely used in many research areas to find a local minimum of a differentiable function.^17^, ^18^, ^19^ We provide the derivatives for the log‐likelihood, ∇l(α), which given in Appendix C for reference. Due to the logarithms within the gradient function, the iterative optimization algorithms are prone to exploding gradients. This often occurs in neural networks.^20^ One way reduce the occurrence of this problem is by using an adaptive step size. We use six different step sizes at each iteration, to reduce the occurrence of this problem for the results in Section 3.

Now we know how to fit the model, we need to perform model selection to decide which and how many terms to include within $\Delta_{t}$. In the model set up, we have two parameters for each sensor plus the constant term. However, some sensors (even the regression on the sensor of interest) may not be important for modeling the probability. In the interest of parsimony we use a stepwise regression selection process to select which sensors to include.

We use a greedy forward selection process, adding a sensor to the model if it improves the model for a certain criterion, a common method of fitting regression models.^21^ We use BIC as it is more conservative criteria than AIC. This is preferable for our application as we want to reduce the computation time as we are fitting the model to each sensor and across customers. We now provide results on different sensors provided by Howz.

## 3 HOWZ DATA EXAMPLE

Due to the categorical nature of the data, it is difficult to identify the accuracy of each estimated probability in comparison to 0‐1 sensor triggering. Especially when there is often a low chance of a sensor triggering. Therefore, to assess the validity of the model, we construct point‐wise quantile intervals over a month of data. The data provided by Howz comprises of two months, January and February. We take data from January to estimate the parameters, $\hat{\alpha }$, using the method described in Section 2. Then, we run the model on February, estimating the one‐step ahead probabilities, using the model parameter estimates from January.

We choose 6 different step sizes, $\left\{5\times 10^{-5},0.01,0.5,1,2.5,4\right\}$ to give a wide range for the adaptive BFGS. For the stopping criterion we set $\left|l(\alpha_{n})\right|<1e-5$ for the nth step in the BFGS and set a maximum number of iterations to be performed as 500.

We take the data from February and simulate an online process, calculating the one‐step ahead estimated probability, using the data gathered up until each future time t.

(1) $$\begin{array}{ll} P(Y_{t+1}|F_{t}) & =E(Y_{t+1}|F_{t})=\frac{\exp({\hat{\Delta }}_{t+1})}{1+\exp({\hat{\Delta }}_{t+1})}, \end{array}$$

(2) $$\begin{array}{ll} \text{where}\;{\hat{\Delta }}_{t+1} & =â+{\hat{\pi }}_{b}\overset{t}{\underset{i=1}{\sum }}{\hat{\phi }}_{b}^{i-1}y_{t-i+1}+\overset{J}{\underset{j=1}{\sum }}{\hat{\tau }}_{j}\overset{t}{\underset{i=1}{\sum }}{\hat{\psi }}_{j}^{i-1}z_{j,t-i+1} \end{array}$$

(3) $$\begin{array}{ll} & \;+\left\{\begin{array}{ll} {\hat{\pi }}_{d}\overset{\left\lfloor \frac{t+1}{96}\right\rfloor }{\underset{i=1}{\sum }}{\hat{\phi }}_{d}^{i-1}\max(y_{t-96i:t-96i+2}),\; & \text{if}\;t+1\geq 96,\\ 0,\; & \text{otherwise.} \end{array}\right. \end{array}$$

The first 96 estimates, the first day, are discarded as burn in. Now for each 15‐minute period in 24 hours, we have around 27 estimated probabilities. While we are aware of the routines present in the data, it would be unrealistic to assume these groups of probabilities were identically distributed, that is, a member of the household follows the same routine at the exact same time each day. Therefore, we use the Poisson binomial distribution to get the quantiles for our estimates. The Poisson binomial distribution is the sum of independent Bernoulli trials which are not enforced to be identically distributed.^22^ This provides an interval closer to reality as we cannot assume the different probabilities are identically distributed but they are likely to be similar across days.

Let the estimated probabilities be $\left\{{\hat{p}}_{i,w}\right\}$ for i=1,…,27 and w be the time period in the day, that is, 1,…,96. Then the distribution can be expressed as,

$$X_{w}=\overset{n}{\underset{i=1}{\sum }}X_{i,w}\;\text{with}\;X_{i,w}∼\text{Bern}(p_{i,w}).$$

The mean and variance are

$$\mu =\overset{n}{\underset{i=1}{\sum }}p_{i,w}\;\text{and}\;\sigma^{2}=\overset{n}{\underset{i=1}{\sum }}p_{i,w}(1-p_{i,w}).$$

The package poibin in R^23^ provide estimates of the quantiles of this distribution (2.5% and 97.5%). To compare to the observed data, we take the binary time series and for each time period, 1,…,96, sum the occurrences of the sensor of interest over February. If the model is appropriate we would expect the empirical sum to lie within the predicted quantile interval from the model.

We show one example from 2 different households; one with 10 sensors and one with 4 sensors. For more examples, see Appendix D.

The following two households are chosen from a range of households provided by Howz as they show different features. We seek to demonstrate that the method works where households have a small or large number of additional sensors and selected households that show sensors that have some “spikes” rather than a flat average behavior over the day (like household 3 in the Appendix). These are typical examples of households within the wider dataset provided to us.

### 3.1 Household 1

We present a household monitored by Howz, including 10 sensors: bedroom, lounge, bathroom, hallway, kitchen, fridge door, kettle, front door, back door, and toaster. We model the bedroom sensor, using the model selection process in Section 2. The model selection indicates that the hallway and front door sensors are informative for modeling the bedroom sensor, along with the bedroom sensor itself and the daily seasonal component. The parameter estimates are given in Table 1.

**TABLE 1:** Parameter estimates from fitting the model in Section 2 to household 1, bedroom sensor

α	$\pi_{b}$	$\phi_{b}$	$\tau_{\mathrm{Hallway}}$	$\psi_{\mathrm{Hallway}}$	$\tau_{\mathrm{FrontDoor}}$	$\psi_{\mathrm{FrontDoor}}$	$\pi_{d}$	$\phi_{d}$
−3.009	1.578	−0.040	0.683	0.547	−0.403	0.926	0.322	0.824

**FIGURE 3:**
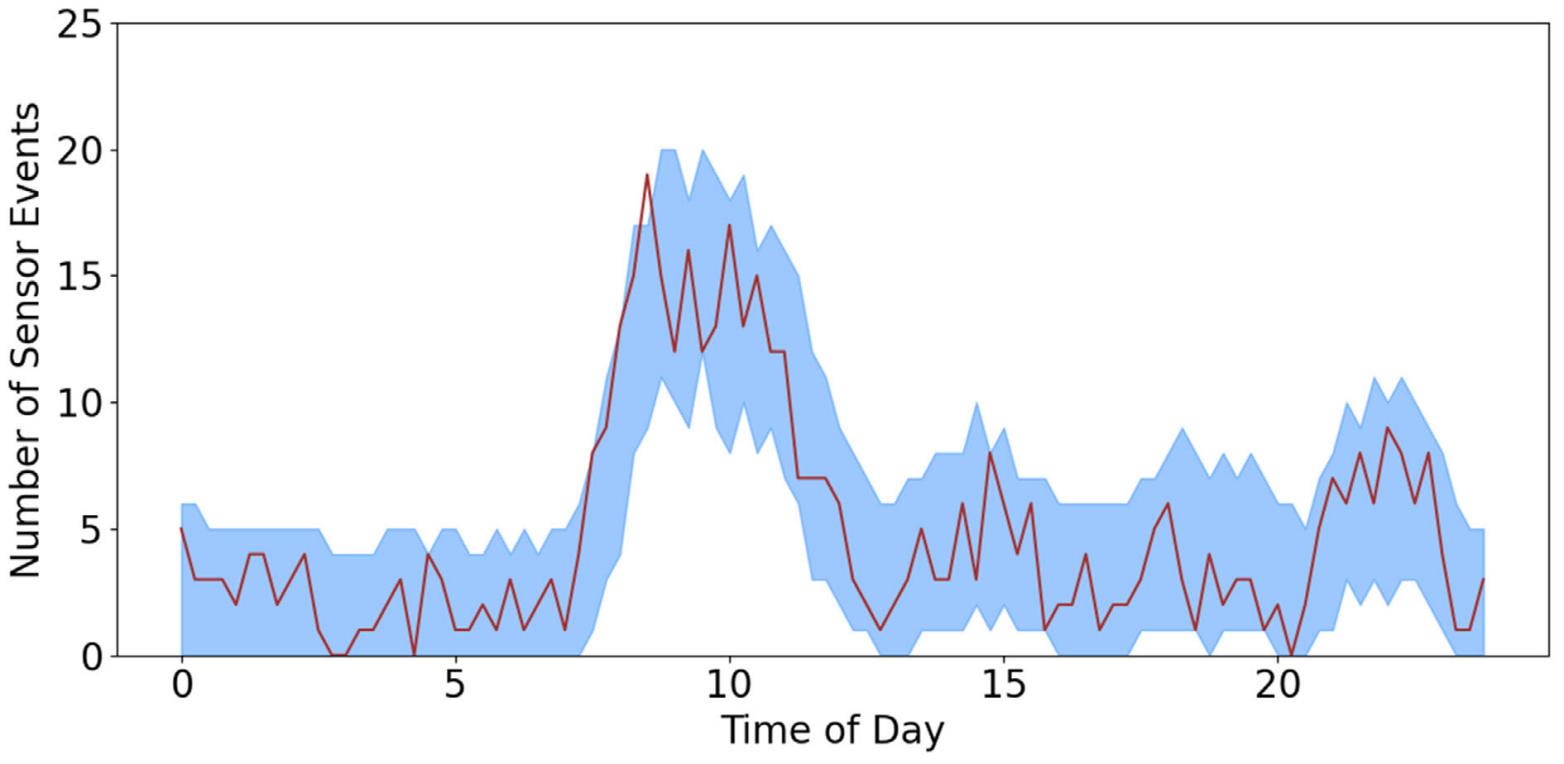
Number of events seen over 27 days (line) with the predicted 95% quantile interval (shaded band) for the bedroom sensor

Figure 3 depicts the 95% quantile band alongside the number of true events over the 27 days which shows that the method adapts well to the change in the number of events over the day. The quantile band follows the clear peaks at the beginning of the households day and the end, between 9 am and 12 pm and after 8 pm, respectively. It is also able to follow the lower level activity throughout the afternoon. We have 96 points and 1 falls outside the quantile interval indicating that the false positive rate is controlled.

Table 1 shows that the bedroom sensor has a large positive spike coefficient, meaning that if we see the bedroom sensor in the previous time period we are more likely to see it in the next time period. Conversely, if we see the Front door sensor, we are less likely to see the bedroom sensor triggering in the next 15 minutes.

The computational time for this method is split into three parts: model selection, parameter estimation, and probability estimates. In practice, the model selection and parameter estimation will be run offline. The model selection for the bedroom sensor in this example took 424 seconds (machine with 2.8 GHz processor and 16 GB RAM) without parallelization. The estimation of the parameters after model selection took around 21 seconds with 40 iterations of the adaptive BFGS (where each iteration checked 6 step sizes). Finally it took around 2 seconds to estimate the probabilities for all of February. In practice, these probabilities will be calculated sequentially taking very little computational time.

### 3.2 Household 2

Consider a different household with four sensors: bedroom, hallway, kettle, and microwave. To test how well this method works with varying amounts of data, this household has fewer sensors so will have vastly fewer covariates (the sensors being triggered). We wish to estimate the quantile interval over a day for the bedroom sensor in February. The model selection returns that kettle is informative with the autoregressive term and the daily seasonal term. The method estimates the parameters as in Table 2.

**TABLE 2:** Parameter estimates from fitting the model in Section 2 to household 2, bedroom sensor

α	$\pi_{b}$	$\phi_{b}$	$\tau_{\mathrm{Kettle}}$	$\psi_{\mathrm{Kettle}}$	$\pi_{d}$	$\phi_{d}$
−3.325	0.770	0.170	−2.124	0.310	0.637	0.817

**FIGURE 4:**
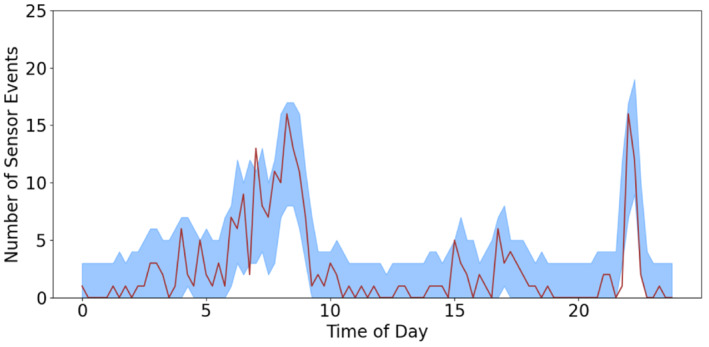
Number of events seen over 27 days (line) with the predicted 95% quantile interval (shaded band) for the bedroom sensor, household 2

Using these parameters, we estimate the quantile interval displayed in Figure 4 and see again it follows the peaks seen in the total number of events. We can see this household tends to get up between 6 and 9 am, with the user more likely to be active in the bedroom closer to 9 am. The quantile band is able to follow this noisy upwards trend in the morning and steep decline around 9 am, as well as the large spike around 10 pm for the end of the households day. Again the method is shown to control the false positive rate well with 3 outside the band.

It is interesting to note that the constant term, a, is negative for all of the examples. This is due to balancing the rate and spike parameters, ensuring that the baseline probability of a sensor being triggered is small if none of the sensors chosen from the model selection have triggered. We also see from Table 2 that the kettle sensor has a negative spike parameter. This tells us, if we see the kettle in the previous 15 minutes we are unlikely to see the bedroom sensor. However for the fridge door and seasonal component, if we see it in the previous 15 minutes we are likely to see it in the next 15 minutes.

Household 2 has a smaller number of sensors, hence the computational time for this example is reduced. It took around 72 seconds for the model selection, 6 seconds for the parameter estimation with 25 iterations and 1 second to calculate the estimated probabilities for February.

## 4 COMPARISON TO LOGISTIC REGRESSION

Next we compare to the logistic regression to further test the validity of the model. We define the linear predictor, $\Delta_{t}$, using the same notation,

$$\Delta_{t}=a+\pi_{b}y_{t-1}+\overset{J}{\underset{j=1}{\sum }}\tau_{j}z_{j,t-1}+\pi_{d}\max(y_{t-96-1:t-96+1}).$$

The package statsmodel ^24^ in python provides the estimated one‐step ahead estimated probabilities. These probabilities can be used to form similar quantile intervals as the examples in Section 3.

### 4.1 Howz data examples

Using these probabilities, we present how many points fall outside the quantile interval for the Howz data examples in Section 3 and Appendix D. Table 3 shows the Bernoulli autoregressive method has considerably fewer points outside the 95% quantile interval for all households. See Appendix D for the other household examples.

**TABLE 3:** Comparison between logistic model and the Bernoulli autoregressive model with columns (left to right): Household number, type of sensor being predicted, how many points lie outside of the quantile interval for the logistic regression model and the Bernoulli autoregressive model

		Logistic regression model	Bernoulli autoregressive model
Household	Sensor	Outside QI	Outside QI
1	Bedroom	9	1
1	Fridge door	8	2
2	Bedroom	7	3
2	Kettle	4	0
2	Hallway	12	5
2	Microwave	6	0
3	Kettle	4	0

**FIGURE 5:**
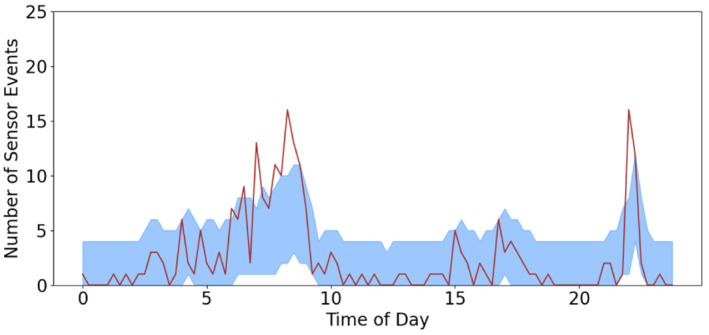
Number of events seen over 27 days (line) with the predicted 95% quantile interval (shaded band) for the bedroom sensor, household 2, using logistic regression model

For a more direct visual comparison we present the quantile interval figure for the bedroom sensor from household 2 using the logistic model, in Figure 5. Here it is clear to see the benefits of the Bernoulli autoregressive model, Figure 4, over the logistic model. When there are sharp spikes and dips (in the morning and evening), the logistic regression model struggles to follow the behavior. It also struggles with fast changes in behavior, for example, between 5 and 10 am, the logistic method slowly trends upwards. In comparison, in Figure 4, we can see the method is following the behavior much better. Further, it can be seen that it is able to follow the sharp spikes in behavior well.

### 4.2 Simulating from the Bernoulli autoregressive model

Turning now to consider performance in the simulation setting, we can simulate a new sensor X to compare our method to the logistic regression. To set up the simulation, we imagine a new sensor (X) within household 2. Sensor X is dependent on the bedroom sensor, the sensor itself and the seasonal component. To simulate from the Bernoulli autoregressive model, we specify the parameters in Table 4.

**TABLE 4:** Parameters to simulate sensors

Model	α	$\pi_{b}$	$\phi_{b}$	$\tau_{\mathrm{Bedroom}}$	$\psi_{\mathrm{Bedroom}}$	$\pi_{d}$	$\phi_{d}$
Bernoulli autoregressive	−3.3	0.3	0.5	0.5	0.9	0.4	0.8
Logistic	−2.8	0.8	‐	1.2	‐	1.4	‐

In context, the positive spike parameters mean we are more likely (to varying degrees) to see sensor X triggered if we have seen the bedroom and the sensor itself recently, as well as seeing the sensor at a similar time the day before. Using the bedroom sensor observations from household 2, we can simulate January and February for sensor X using our method. We estimate the parameters chosen above on the January data for both models. Similar to Section 3, we then use these model estimates to get the predicted probability estimates for February. We repeat this experiment 500 times to gather the mean number of points outside the quantile intervals.

**FIGURE 6:**
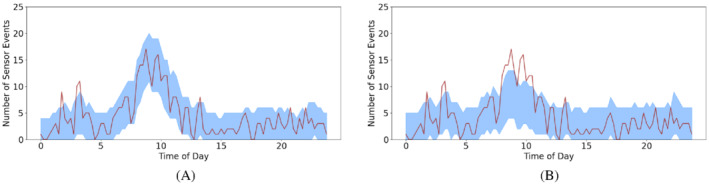
Number of events seen over 27 days (line) with the predicted 95% quantile interval (shaded band) for sensor X. (A) Bernoulli autoregressive model and (B) logistic regression model

Figure 6A,B shows one realization from each model, we can see that our method is better able to follow the large spike between 5 am and 1 pm. There are 6 points outside the quantile interval for our method vs 12 in the logistic model. Overall from the simulations, the Bernoulli model has a mean of 2.26 outside the quantile interval whereas the logistic model has a mean of 10.32. This shows that the Bernoulli model is better able to predict the simulated data in this example.

### 4.3 Simulating from the logistic model

Using the same set up as Section 4.2, we instead simulate from the logistic regression model using alternative parameters in Table 4 for a new sensor Y. Again we repeat the experiment 500 times.

**FIGURE 7:**
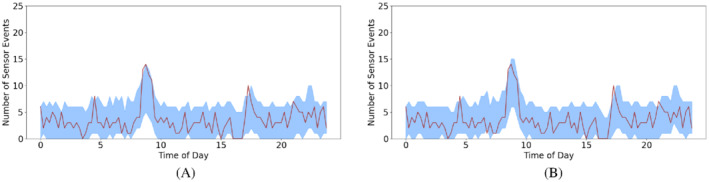
Number of events seen over 27 days (line) with the predicted 95% quantile interval (shaded band) for sensor Y. (A) Bernoulli autoregressive model and (B) logistic regression model

From the simulations, the Bernoulli model has a mean of 2.97 outside the quantile interval whereas the logistic model has a mean of 2.99. This confirms our method is able to predict the behavior as well as the logistic regression. For an example of what sensor Y could look like, Figure 7A,B shows that both methods are able to follow the spike just before 10 am and both follow the true number events seen well throughout the day. There are 3 points outside the quantile interval for our method vs 5 in the logistic regression. Overall it is clear to see that our method performs well for our application, when simulating from our model and when using data simulated from the logistic regression model.

## 5 CONCLUSION AND FUTURE RESEARCH

In this article, we have presented a method which is able to predict the one step ahead probability of a sensor being triggered given other household sensors. Using model selection, it is able to choose which sensors are of importance to the estimation and provide a good estimate on the Howz data as seen in Section 3. After the initial model selection and parameter estimation, it is computationally efficient enough to predict online or at the end of every day.

Using quantile intervals we have demonstrated the validity of the method in predicting the probability of sensor activation. The number of events seen outside the quantile band are controlled well, with the results able to follow the spikes and dips throughout the 24‐hour period. When comparing to the logistic regression, we see that the method is able to follow the spikes within the data better with fewer outside the quantile intervals. The model adapts well to households with a range of different sensors, as well as households with between 2 and 10 sensors. When simulating data from our model, it is obvious that the Bernoulli autoregression model outperforms the logistic regression. We have also demonstrated the Bernoulli autoregression model is also able to perform as well as the logistic regression when simulating data from a logistic regression model.

The next step would be to adapt this to identify when changes in behavior occur on a 15‐minute/daily basis. Currently, the data presented is assumed to be regular for the household, that is, no significant changes to households routines and behaviors. However, this method can currently provide information and alerts on a monthly scale, when a household is outside the quantile intervals, indicating a potential change. In this article, we have focused on predicting each sensor separately, our approach could also be extended to look at the multivariate fit of the sensors to further consider the interactions between the sensors.

## Figures and Tables

**FIGURE D1:**
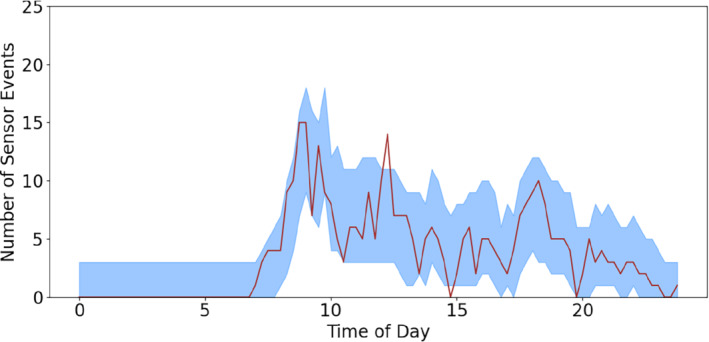
Number of events seen over 27 days (line) with the predicted 95% quantile interval (shaded band) for the fridge door sensor

**FIGURE D2:**
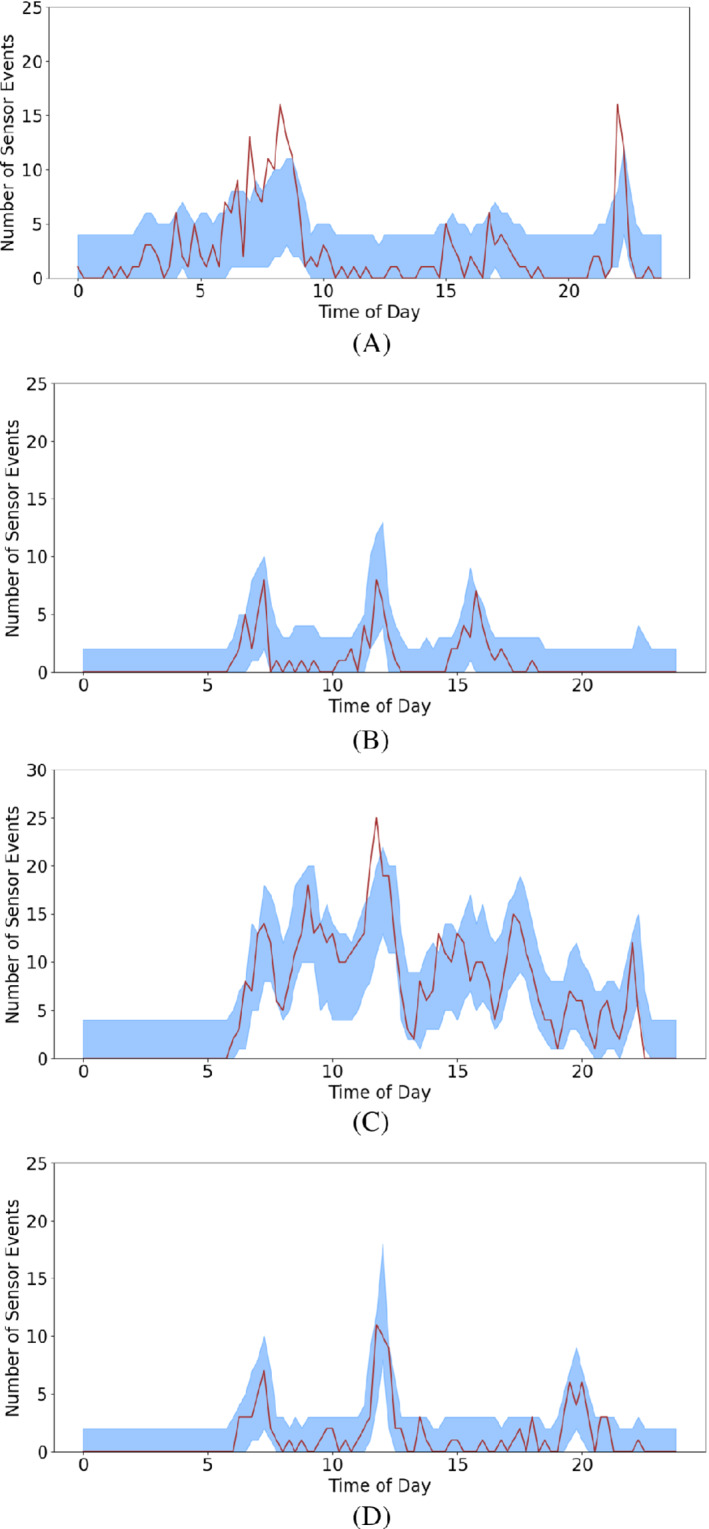
Number of events seen over 27 days (line) with the predicted 95% quantile interval (shaded band) for each sensor. Please note that these were modeled individually. (A) Bedroom sensor, (B) kettle sensor, (C) hallway sensor, and (D) microwave sensor

**FIGURE D3:**
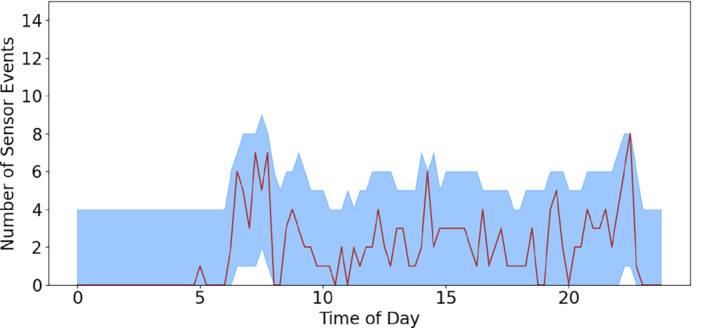
Number of events seen over 27 days (line) with the predicted 95% quantile interval (shaded band) for the kettle sensor

## Data Availability

The data that support the findings of this study are available from Howz. Restrictions apply to the availability of these data, which were used under license for this study. Data are available from the industrial authors with the permission of Howz.

## APPENDIX A SIMPLIFYING THE LINK FUNCTION

Assuming $b_{0}=0$ and $y_{0}=0$ to initialize we can simplify $b_{t}$ to;

$$\begin{array}{llll} b_{t} & =\phi_{b}b_{t-1}+\pi_{b}y_{t-1}\; & & \\ & =\phi_{b}\left[\phi_{b}b_{t-2}+\pi_{b}y_{t-2}\right]+\pi_{b}y_{t-1}\; & & \\ & =\phi_{b}^{t}b_{0}+\left[\phi_{b}^{t-1}\pi_{b}y_{b,0}+\phi_{b}^{t-2}\pi_{b}y_{1}+\cdots +\phi_{b}\pi_{b}y_{t-2}+\pi_{b}y_{t-1}\right]\; & & \\ & =\pi_{b}\left[\phi_{b}^{t-2}y_{1}+\cdots +\phi_{b}y_{t-2}+y_{t-1}\right]\; & & \\ & =\pi_{b}\overset{t-1}{\underset{i=1}{\sum }}\phi_{b}^{i-1}y_{t-i}.\; & & \end{array}$$

Similarly, assuming $c_{j,0}=0$ and $z_{j,0}=0$,

(A1)
$$c_{j,t}=\tau_{j}\overset{t-1}{\underset{i=1}{\sum }}\psi_{j}^{i-1}z_{j,t-i}.$$

If we set $y_{-1}=0$, $y_{0}=0$ and consider t≥96,

$$\begin{array}{llll} d_{t} & =\phi_{d}d_{t-96}+\pi_{d}\max(y_{t-96-1:t-96+1})\; & & \\ & =\phi_{d}\left[\phi_{d}d_{t-96(2)}+\pi_{d}\max(y_{t-96*2-1:t-96*2+1})\right]+\pi_{b}\max(y_{t-96-1:t-96+1})\; & & \\ & =\pi_{d}\left[\phi_{d}^{\left\lfloor \frac{t}{96}\right\rfloor }\max(y_{t-96\left\lfloor \frac{t}{96}\right\rfloor -1:t-96\left\lfloor \frac{t}{96}\right\rfloor +1})+\cdots \;\right.\; & & \\ & \left.\;+\phi_{d}\max(y_{t-96*2-1:t-96*2+1})+\max(y_{t-96-1:t-96+1})\right]\; & & \\ & =\pi_{d}\overset{\left\lfloor \frac{t}{96}\right\rfloor }{\underset{i=1}{\sum }}\phi_{d}^{i-1}\max(y_{t-96i-1:t-96i+1}),\;\text{for}\;t\geq 96.\; & & \end{array}$$

Therefore, $\Delta_{t}$ and $p_{t}$ can be expressed as,

$$\begin{array}{llll} \Delta_{t} & =a+\pi_{b}\overset{t-1}{\underset{i=1}{\sum }}\phi_{b}^{i-1}y_{t-i}+\overset{J}{\underset{j=1}{\sum }}\tau_{j}\overset{t-1}{\underset{i=1}{\sum }}\psi_{j}^{i-1}z_{j,t-i}\; & & \\ & \;+\left\{\begin{array}{cc} \pi_{d}\overset{\left\lfloor \frac{t}{96}\right\rfloor }{\underset{i=1}{\sum }}\phi_{d}^{i-1}\max(y_{t-96i-1:t-96i+1}),\; & \text{if}\;t\geq 96,\\ 0,\; & \text{otherwise.} \end{array}\right.\; & & \\ p_{t} & =\frac{\exp(\Delta_{t})}{1+\exp(\Delta_{t})}.\; & & \end{array}$$

## APPENDIX B DERIVATION OF LOG‐LIKELIHOOD

Given the parameter set, $\alpha =\{a,\pi_{b},\phi_{b},\tau_{1},\psi_{1},\ldots ,\tau_{j},\psi_{j},\pi_{d},\phi_{d},\}$, with $\left\{y_{t}\right\}$ from a Bernoulli distribution, we can express the Likelihood as,

$$\begin{array}{llll} L\left(\alpha |{\left\{y_{t}\right\}}_{t=1}^{T}\right) & =\overset{T}{\underset{t=1}{\prod }}p_{t}^{y_{t}}{\left(1-p_{t}\right)}^{1-y_{t}}\; & & \\ & =\overset{T}{\underset{t=1}{\prod }}{\left(\frac{\exp(\Delta_{t})}{1+\exp(\Delta_{t})}\right)}^{y_{t}}\times {\left(1-\frac{\exp(\Delta_{t})}{1+\exp(\Delta_{t})}\right)}^{1-y_{t}}\; & & \\ & =\overset{T}{\underset{t=1}{\prod }}\frac{\exp(y_{t}\Delta_{t})}{{\left(1+\exp(\Delta_{t})\right)}^{y_{t}}{\left(1+\exp(\Delta_{t})\right)}^{1-y_{t}}}\; & & \\ & =\overset{T}{\underset{t=1}{\prod }}\frac{\exp(y_{t}\Delta_{t})}{1+\exp(\Delta_{t})}.\; & & \end{array}$$

We now consider the log‐likelihood in the estimation of α, to find

$$\begin{array}{llll} l(\alpha |{\left\{y_{t}\right\}}_{t=1}^{T}) & =\log\left(\overset{T}{\underset{t=1}{\prod }}\frac{\exp(y_{t}\Delta_{t})}{1+\exp(\Delta_{t})}\right)\; & & \\ & =\overset{T}{\underset{t=1}{\sum }}\log\left(\frac{\exp(y_{t}\Delta_{t})}{1+\exp(\Delta_{t})}\right)\; & & \\ & =\overset{T}{\underset{t=1}{\sum }}y_{t}\Delta_{t}\;-\;\overset{T}{\underset{t=1}{\sum }}\log\left(1+\exp(\Delta_{t})\right).\; & & \end{array}$$

## APPENDIX C GRADIENT OF THE LOG‐LIKELIHOOD

We define the gradient of the log‐likelihood as

$$\nabla l\left(\alpha \right)=\left\{\frac{\partial l\left(\alpha \right)}{\partial a},\frac{\partial l\left(\alpha \right)}{\partial \pi_{b}},\frac{\partial l\left(\alpha \right)}{\partial \phi_{b}},\frac{\partial l\left(\alpha \right)}{\partial \tau_{1}},\frac{\partial l\left(\alpha \right)}{\partial \psi_{1}},\ldots ,\frac{\partial l\left(\alpha \right)}{\partial \tau_{j}},\frac{\partial l\left(\alpha \right)}{\partial \psi_{j}},\frac{\partial l\left(\alpha \right)}{\partial \pi_{d}},\frac{\partial l\left(\alpha \right)}{\partial \phi_{d}}\right\}.$$

Assuming $y_{-1}=0$, $y_{0}=0$, and $z_{j,0}=0$ for j=1,…,J. We can find the partial derivatives of the log‐likelihood as

$$\begin{array}{llll} \frac{\partial l\left(\alpha \right)}{\partial a} & =\overset{T}{\underset{t=1}{\sum }}y_{t}-\overset{T}{\underset{t=1}{\sum }}\frac{\exp\left(\Delta_{t}\right)}{1+\exp\left(\Delta_{t}\right)},\; & & \\ \frac{\partial l\left(\alpha \right)}{\partial \pi_{b}} & =\overset{T}{\underset{t=1}{\sum }}y_{t}\overset{t-1}{\underset{i=1}{\sum }}\phi_{b}^{i-1}y_{t-i}-\overset{T}{\underset{t=1}{\sum }}\frac{\exp\left(\Delta_{t}\right)}{1+\exp\left(\Delta_{t}\right)}\overset{t-1}{\underset{i=1}{\sum }}\phi_{b}^{i-1}y_{t-i},\; & & \\ \frac{\partial l\left(\alpha \right)}{\partial \phi_{b}} & =\pi_{b}\overset{T}{\underset{t=1}{\sum }}y_{t}\overset{t-1}{\underset{i=2}{\sum }}\left(i-1\right)\phi_{b}^{i-2}y_{t-i}-\pi_{b}\overset{T}{\underset{t=1}{\sum }}\frac{\exp\left(\Delta_{t}\right)}{1+\exp\left(\Delta_{t}\right)}\overset{t-1}{\underset{i=2}{\sum }}\left(i-1\right)\phi_{b}^{i-2}y_{t-i}.\; & & \end{array}$$

For other sensors j=1,…,J:

$$\begin{array}{llll} \frac{\partial l\left(\alpha \right)}{\partial \tau_{j}} & =\overset{T}{\underset{t=1}{\sum }}y_{t}\overset{t-1}{\underset{i=1}{\sum }}\psi_{j}^{i-1}z_{j,t-i}-\overset{T}{\underset{t=1}{\sum }}\frac{\exp\left(\Delta_{t}\right)}{1+\exp\left(\Delta_{t}\right)}\overset{t-1}{\underset{i=1}{\sum }}\psi_{j}^{i-1}z_{j,t-i},\; & & \\ \frac{\partial l\left(\alpha \right)}{\partial \psi_{j}} & =\tau_{j}\overset{T}{\underset{t=1}{\sum }}y_{t}\overset{t-1}{\underset{i=2}{\sum }}\left(i-1\right)\psi_{j}^{i-2}z_{j,t-i}-\tau_{j}\overset{T}{\underset{t=1}{\sum }}\frac{\exp\left(\Delta_{t}\right)}{1+\exp\left(\Delta_{t}\right)}\overset{t-1}{\underset{i=2}{\sum }}\left(i-1\right)\psi_{j}^{i-2}z_{j,t-i}.\; & & \end{array}$$

For the seasonal component with t≥96:

$$\begin{array}{llll} \frac{\partial l\left(\alpha \right)}{\partial \pi_{d}} & =\overset{T}{\underset{t=1}{\sum }}y_{t}\overset{\left\lfloor \frac{t}{96}\right\rfloor }{\underset{i=1}{\sum }}\phi_{d}^{i-1}\max\left(y_{t-96i-1:t-96i+1}\right)\; & & \\ & \;-\overset{T}{\underset{t=1}{\sum }}\frac{\exp\left(\Delta_{t}\right)}{1+\exp\left(\Delta_{t}\right)}\overset{\left\lfloor \frac{t}{96}\right\rfloor }{\underset{i=1}{\sum }}\phi_{d}^{i-1}\max\left(y_{t-96i-1:t-96i+1}\right),\; & & \\ \frac{\partial l\left(\alpha \right)}{\partial \phi_{d}} & =\pi_{d}\overset{T}{\underset{t=1}{\sum }}y_{t}\overset{\left\lfloor \frac{t}{96}\right\rfloor }{\underset{i=2}{\sum }}\left(i-1\right)\phi_{d}^{i-2}\max\left(y_{t-96i-1:t-96i+1}\right)\; & & \\ & \;-\pi_{d}\overset{T}{\underset{t=1}{\sum }}\frac{\exp\left(\Delta_{t}\right)}{1+\exp\left(\Delta_{t}\right)}\overset{\left\lfloor \frac{t}{96}\right\rfloor }{\underset{i=2}{\sum }}\left(i-1\right)\phi_{d}^{i-2}\max\left(y_{t-96i-1:t-96i+1}\right).\; & & \end{array}$$

## APPENDIX D HOWZ DATA EXAMPLES

### D.1 Household 1

Taking a different sensor from this household, fridge door, the model selection process identifies the hallway, kettle, and lounge sensors as informative but not the fridge door sensor itself. The estimated parameters are given in Table D1 and the prediction interval in Figure D1.

**TABLE D1:** Parameter estimates from fitting the model in Section 2 to household 1, fridge door sensor

α	$\tau_{\mathrm{Hallway}}$	$\psi_{\mathrm{Hallway}}$	$\tau_{\mathrm{Kettle}}$	$\psi_{\mathrm{Kettle}}$	$\tau_{\mathrm{Lounge}}$	$\psi_{\mathrm{Lounge}}$	$\pi_{d}$	$\phi_{d}$
−3.634	0.848	−0.446	1.027	0.084	0.680	0.209	0.392	0.809

Again the method follows the change in number of events well with two events outside the interval. For this sensor, we see the household uses the fridge in the morning, around lunch time and then in the evening with low level usage throughout the day.

### D.2 Household 2

Household 2 has four sensors: bedroom, hallway, kettle, and microwave. In Section 3.2, we present the bedroom sensor. We now give the other sensors in household 2, starting with the kettle sensor, which after performing the model selection chooses the hallway and bedroom but not the kettle sensor itself. The estimates of the parameters are given in Table D2. The estimation for the 95% quantile interval for February is given in Figure D2A which shows the household has three clear time periods throughout the day the kettle is used: morning, lunch, and afternoon. The method follows the peaks well even with the reduced amount of data due to the kettle being used infrequently.

**TABLE D2:** Parameter estimates from fitting the model in Section 2 to household 2, kettle sensor

α	$\tau_{\mathrm{Hallway}}$	$\psi_{\mathrm{Hallway}}$	$\tau_{\text{Bedroom}}$	$\psi_{\text{Bedroom}}$	$\pi_{d}$	$\phi_{d}$
−4.093	0.807	−0.191	0.765	0.044	0.530	0.871

Next we give the hallway sensor. The model selections choose the kettle, bedroom, autoregressive term, and seasonal component. The estimates of the parameters are given in Table D3. The estimation for the 95% quantile interval for February is given in Figure D2B which shows the household has behavior throughout the day. The method follows the peaks well, however, struggles a little to capture the midday spike.

**TABLE D3:** Parameter estimates from fitting the model in Section 2 to household 2, hallway sensor

α	$\pi_{b}$	$\phi_{b}$	$\tau_{\mathrm{Kettle}}$	$\psi_{\mathrm{Kettle}}$	$\tau_{\text{Bedroom}}$	$\psi_{\text{Bedroom}}$	$\pi_{d}$	$\phi_{d}$
−3.135	1.306	−0.207	0.653	0.933	0.623	0.365	0.447	0.798

Finally, we give the microwave sensor, the model selection chooses the kettle, hallway, and seasonal term (see Table D4 for the parameter estimates). The estimation for the 95% quantile interval for February is given in Figure D2C which shows the household has three clear microwave usage times throughout the day: morning, lunch, and evening. Similar to the kettle sensor, the method follows the peaks well with the reduced amount of data (Table D4).

**TABLE D4:** Parameter estimates from fitting the model in Section 2 to household 2, microwave sensor

α	$\tau_{\mathrm{Kettle}}$	$\psi_{\mathrm{Kettle}}$	$\tau_{\mathrm{Hallway}}$	$\psi_{\mathrm{Hallway}}$	$\pi_{d}$	$\phi_{d}$
−4.263	1.057	−0.705	0.889	−0.244	0.836	0.801

The spike parameters, π and τ, give a good indication of the interactions between the sensors. For example in Table D1, we see the kettle spike parameter is negative, so we are unlikely to see the bedroom sensor if we have just seen the kettle. However, in Table D2, we can see that if we see have seen the bedroom sensor recently we are likely to see the kettle. The kettle and hallway sensors behave differently, looking at the spike parameters in Tables D2 and D3, we can see that seeing the kettle recently means we are likely to see the hallway and vice‐versa. The model selection for the microwave chose the kettle and hallway sensor, with both having positive spike parameters, suggesting seeing the kettle and/or hallway will mean we are more likely to see the microwave. However, neither of those chose the microwave sensor during model selection. While these differing relationships will be unique to the household, it is interesting to see the effect of users interactions with the sensors placed throughout the house.

### D.3 Household 3

We present a household which has two sensors: front door and kettle. This household has little information, so is a good test for how the method works on the opposite end of the spectrum from household 1.

We present the results from the kettle sensor. The model selection process selected the kettle itself and the seasonal component. The parameter estimates are given in Table D5.

**TABLE D5:** Parameter estimates from fitting the model in Section 2 to household 3, kettle sensor

α	$\pi_{b}$	$\phi_{b}$	$\pi_{d}$	$\phi_{d}$
−2.990	0.159	−0.795	0.322	0.882

Figure D3 shows that even when the sensor is infrequently used and there is little information from the rest of the house, the method is still able to follow the number of true events seen well. Specifically, even with small peaks, the quantile band shifts upwards.
